# ﻿*Lysimachia barcae* (Primulaceae), a new endemic shrub from Wainiha, Kaua‘i, Hawaiian Islands

**DOI:** 10.3897/phytokeys.265.169710

**Published:** 2025-10-21

**Authors:** Kenneth R. Wood, Warren L. Wagner, Susan Fawcett, Scott Heintzman

**Affiliations:** 1 National Tropical Botanical Garden, 3530 Papalina Road, Kalāheo, HI 96741, USA National Tropical Botanical Garden Kalāheo United States of America; 2 Department of Botany, Smithsonian Institution, PO Box 37012, Washington, DC 20013-7012, USA Smithsonian Institution Washington United States of America; 3 University and Jepson Herbaria, 1001 Valley Life Sciences Building #2465, University of California, Berkeley, CA 94720-2465, USA University of California Berkeley United States of America; 4 Plant Extinction Prevention Program, Pacific Cooperative Unit, University of Hawai‘i, 3060 Eiwa St., Līhu‘e, HI 96766, USA University of Hawai‘i Līhu‘e United States of America

**Keywords:** Discovery, endangered species, Hawaiian flora, plant extinction prevention, primrose family, single-island endemism

## Abstract

A new endemic species of *Lysimachia* from Kaua‘i, Hawaiian Islands, is described and illustrated with notes on its distribution, ecology, conservation status and phylogenetic relationships. A modification to the existing key for Hawaiian *Lysimachia* is provided. *Lysimachia barcae***sp. nov.**, differs from its Hawaiian congeners by its unique combination of mature stems villous to tomentose, petioles 1–3 mm long, leaves ovate, cordate to subcordate or rounded at base, with primary and secondary veins conspicuous, purple-red, raised on abaxial surface, often densely hirsute to pilose and pedicels 35–70 mm long. Only ten mature individuals are known from a single colony along steep, precipitous slopes of Wainiha Valley, Kaua‘i. *Lysimachia barcae* represents a new Critically Endangered (CR) single-island endemic species and is the focus of concerted conservation efforts to prevent its extinction.

## ﻿Introduction

*Lysimachia* L. (Primulaceae) is a diverse cosmopolitan genus that contains ca. 288 accepted species (POWO 2025) belonging to the tribe Lysimachieae Reich. Its centre of diversity is in southwest China and further ranges throughout temperate and subtropical regions of the Northern Hemisphere, but is also found in tropical mountain regions of Africa, Australia, South America and Southeast Asia (Wagner et al. 1990, 1999; Zhang et al. 2024; POWO 2025; Quan et al. 2025).

In the Hawaiian Islands, *Lysimachia* is presently represented by 13 endemic, one indigenous and one naturalised introduced species. The endemic species are in subgen. Lysimachiopsis (A.Heller) Handel-Mazzetti and are considered to be a monophyletic group (Marr and Bohm 1997; Hao et al. 2004; Yan et al. 2018), characterised by several derived traits and differ from all non-Hawaiian *Lysimachia* in having woody stems (vs. herbaceous), flowers varying from purple to red or burgundy, green with purple base, white, cream-coloured or white with purple-pink tinge (vs. uniformly white or yellow, rarely salmon or blue) and have tetracolporate pollen (vs. tricolporate) (Oh et al. 2013). Many of the Hawaiian taxa are considered extremely rare with eight species listed as federally endangered and 10 single-island endemics (SIE). One species, *L. forbesii* Rock from the Punalu‘u/Kaluanui Region of northeast O‘ahu, is presumed extinct after not being observed since 1934 (Wood et al. 2019). Kaua‘i has the greatest diversity of endemic *Lysimachia* with nine species, including seven SIE, followed by Maui and Moloka‘i each with four species and one SIE, O‘ahu with three species and one SIE and Lana‘i with one multi-island species. The Big Island of Hawai‘i has no record of endemic *Lysimachia*, having only the widely distributed indigenous species, *L. mauritiana* Lam. (see Key to Hawaiian *Lysimachia*).

## ﻿Materials and methods

Access to the type locality was made by helicopter transport. Botanical voucher collections of *Lysimachia barcae* are curated at the PTBG Herbarium with duplicates distributed at BISH, NY and US (see Specimens examined). All photo images were made by the authors unless otherwise noted. All morphological measurements were taken from dried herbarium specimens and field notes and are presented in the descriptions as follows: length × width, followed by units of measurements (mm, cm or m). We assessed the extinction risk for *L. barcae* following the IUCN Red List Categories and Criteria (IUCN 2012, 2024). The extent of occurrence (EOO) and area of occupancy (AOO) was calculated by using ArcMap 10.6.1 (ESRI 2018) in relation to coordinates recorded while collecting herbarium specimens or making field observations. Geographic coordinates have been truncated to protect exact locations from unauthorised access.

Molecular phylogenetic data were generated using the *Angiosperms353* probe set (Johnson et al. 2019; Lichter-Marck et al., in prep; Fawcett et al., in prep). Haplominer (He et al. 2011) was used within the SORTER2 framework (Mendez-Reneau et al. 2023) on the Savio high-performance computing cluster at University of California, Berkeley, to recover chloroplast DNA from off-target reads, which were aligned to a reference plastome of *Lysimachia clethroides* Duby (Genbank accession NC_064345.1) using MAFFT. This yielded an average of 89% coverage across the 155,078 bp plastome for 33 samples representing all endemic Hawaiian *Lysimachia*, except for the extinct *L. forbesii*. A Maximum-Likelihood phylogeny was inferred using IQTree 3 (Wong et al. 2025).

## ﻿Taxonomic treatment

### ﻿ Lysimachia barcae

Taxon classificationPlantaeEricalesPrimulaceae

K.R.Wood & W.L.Wagner sp. nov.

1A93637B-8B82-5BC1-B359-47D81D423950

urn:lsid:ipni.org:names:77370960-1

Figs 1, 2, 3, 4

#### Diagnosis.

*Lysimachia barcae* is morphologically most similar to *L. hillebrandii* Hook.f. ex A.Gray from which it differs by its combination of mature stems villous to tomentose (vs. glabrate), petioles 1–3 mm long (vs. 4–10 mm), leaves cordate to subcordate or rounded at base, veins conspicuously raised on abaxial surface (vs. leaves cuneate to attenuate at base, veins only slightly raised on abaxial surface) and pedicels pendulous, 35–70 mm long (vs. erect, 12–45 mm).

#### Type.

**USA.** • **Hawaiian Islands, Kaua‘i**: Hanalei District, Wainiha, 22.093, -159.533, 1150 m alt., 15 May 2025 (fl. & fr.), *K.R. Wood, S. Heintzman & S. Deans 19754* (holotype: PTBG 1000099430!; isotypes: BISH!, US!).

#### Description.

***Shrubs***, sprawling to 3.5 m long, 5- to 15-branched; diameter of lower stems ca. 1 cm near base, young and old stems terete, reddish-brown villous to tomentose. ***Leaves*** well-spaced, alternate, firm-chartaceous, ovate, rarely lanceolate, blade (35–)40–70 × (18–)20–45 mm, leaves abaxially and adaxially sparsely reddish-brown hirsute to pilose, often more densely so on veins, primary and secondary veins prominent on abaxial surface, purple-red, tertiary veins pellucid, margins entire, flat to weakly revolute, apex acuminate to attenuate, occasionally falcate, base cordate to subcordate or rounded, petioles 1–3 mm long, reddish-brown tomentose or pilose. ***Flowers*** solitary in leaf axils, 6- to 7-merous, pedicels slender, pendulous, 35–40 mm long, elongating to 60–70 mm long in fruit, with pilose hairs light tan to rusty; calyx 6- to 7-lobed, lobes persistent, lanceolate to broadly lanceolate, 5–6 × 2–3 mm, pilose to strigose; corolla campanulate, lobes obovate, 10–14 × 5–7 mm, purple, tinged yellowish on margins, veins dark purple, outer and inner surface glandular-punctate; filaments connate from base to 1/3 or 1/2 their length, 5–6 mm long, dark purple, anthers 1.2–1.4 mm long; stigma capitate, 1 × 1 mm, yellow-green, style 7–8 mm long, dark purple, ovary superior, glabrous, subglobose, ca. 2 × 2 mm, dark purple. ***Capsules*** subglobose, thick and woody, 6–9 mm long, dehiscent. ***Seeds*** dark brown, irregularly rhomboid, angled, 1–1.2 mm long.

**Figure 1.**
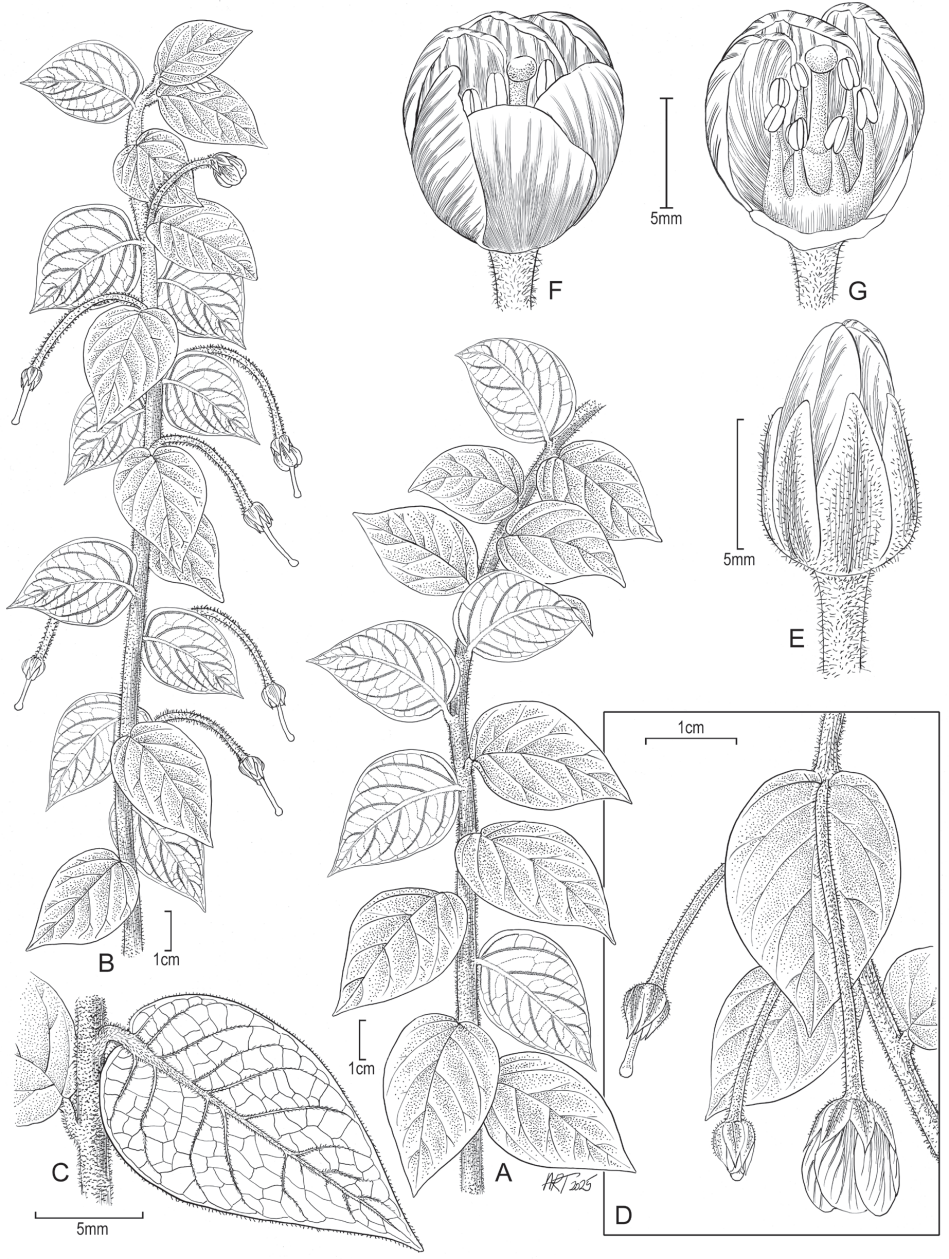
*Lysimachia barcae*. A. Lower portion of habit; B. Upper portion of habit with flowers and immature capsules; C. Abaxial view of leaf blade with petiole; D. Node with pendulous flower and adaxial view of leaf; E. Flower bud; F. Flower showing petals with sepals removed; G. Flower with several petals removed showing stamens and pistil. Drawn from: A. *Wood et al. 19754*, holotype (PTBG); B. Field photo, 15 May 2025, Wainiha, *Wood et al. 19754*; C. Field photo, 19 January 2016, Wainiha, *Wood et al. 16725*; D. Field photo, 15 May 2025, Wainiha, *Wood 19754*; E. Field photo, 19 January 2016, Wainiha, *Wood et al. 16725*; F, G. Nursery photos by Tim Kroessig from plants grown from seed of paratype *Wood, Perlman & Kishida 16725* (Illustration by Alice Tangerini).

#### Additional specimens examined

**(paratypes). USA. Hawaiian Islands, Kaua‘i: Hanalei District**, • **Wainiha**, 1158 m alt., 19 Jan 2016 (fl.), *Wood, Perlman & Kishida 16724* (BISH, PTBG) • loc. cit., 1158 m alt., 19 Jan 2016 (fr.), *Wood, Perlman & Kishida 16725* (BISH, NY, PTBG, US).

**Figure 2.**
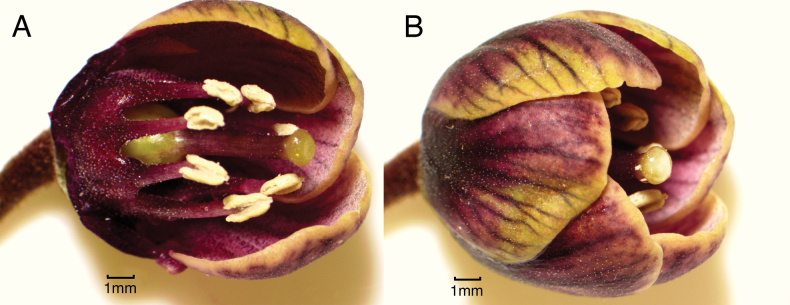
*Lysimachia barcae*. A. Flower with several petals and sepals removed showing pistil and stamens with glands on staminal ring and filaments; B. Flower with petals intact and sepals removed showing pigmentation of petals. A, B. From plants grown from seed of paratype *Wood, Perlman & Kishida 16725* (BISH, NY, PTBG, US). Photos by Tim Kroessig.

#### Phenology.

Due to its extreme isolation, *Lysimachia barcae* has only been observed and vouchered on two occasions, January 2016 and May 2025 and, during those visits, plants were with both flower and fruit.

**Figure 3.**
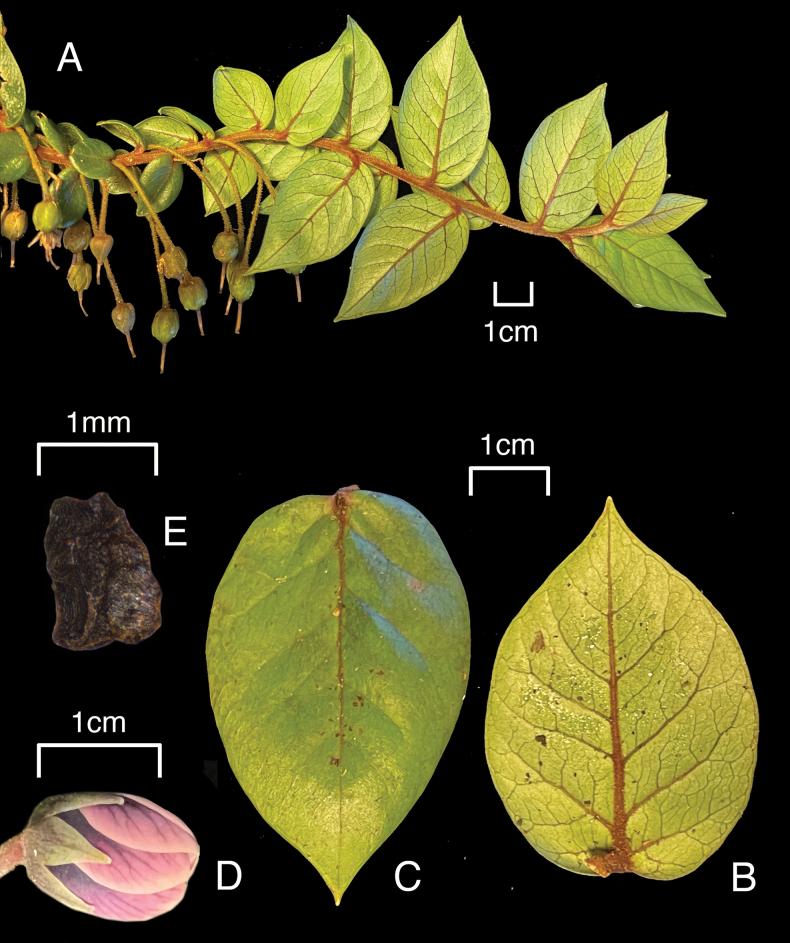
*Lysimachia barcae*. A. Branch, showing pendent immature capsules; B. Leaf, abaxial surface; C. Leaf, adaxial surface; D. Flower, early anthesis; E. Irregularly rhomboid seed. A–D. From *Wood et al. 19754*, holotype (PTBG) E. From *Wood et al. 16725*.

**Figure 4.**
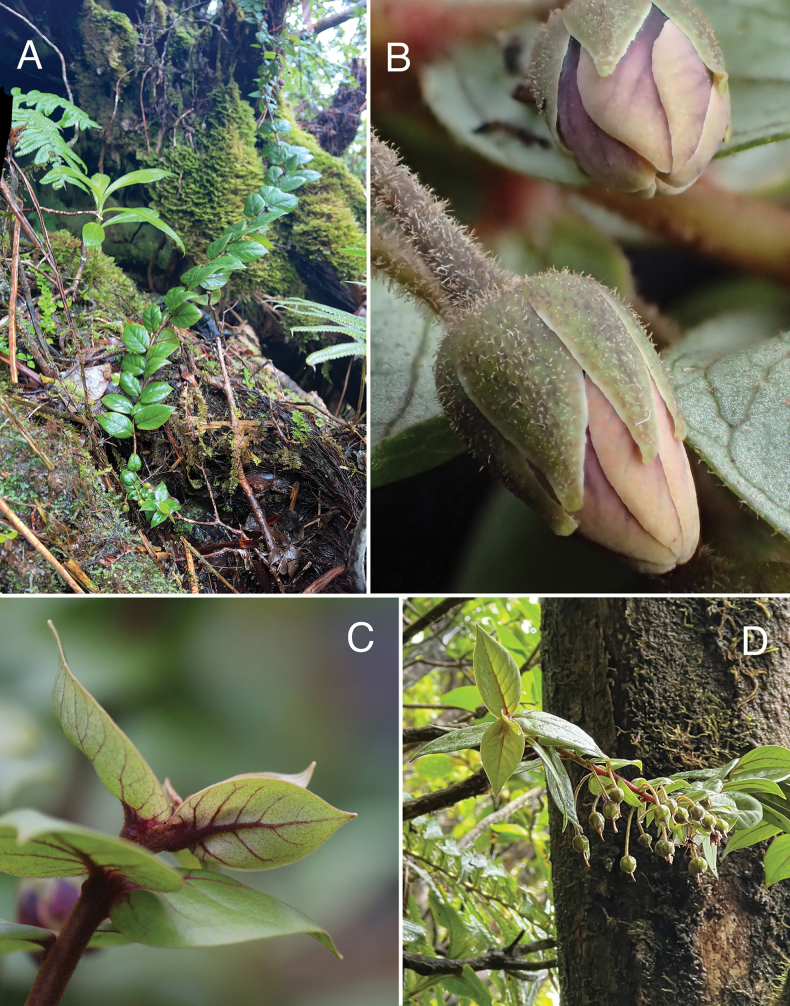
*Lysimachia barcae*. A. Sprawling growth habit; B. Floral buds showing indument on calyx lobes and pedicel; C. Young leaves showing prominent purple-red primary and secondary veins; D. Sprawling habit showing pendent pedicels and immature fruit. A, D. Field photos, 15 May 2025, Wainiha, *Wood et al. 19754*, holotype (PTBG); B. Field photo, 19 January 2016, Wainiha, *Wood et al. 16725*; C. Photo by Tim Kroessig from plant grown from seed of paratype *Wood, Perlman & Kishida 16725* (BISH, NY, PTBG, US).

#### Etymology.

*Lysimachia* is named for Lysimachus (ca. 361–281 BC), King of Thrace and is derived from the Greek *lysis* (release from) and *mache* (strife) (Wagner et al. 1990). The species epithet honours its discoverer, Nicolai Barca, Field Coordinator for The Nature Conservancy of Hawai‘i, Kaua‘i Program, in recognition of his many years of keen observations, hard work and dedication protecting the native forests of Kaua‘i.

#### Affinities.

**Figure 5.**
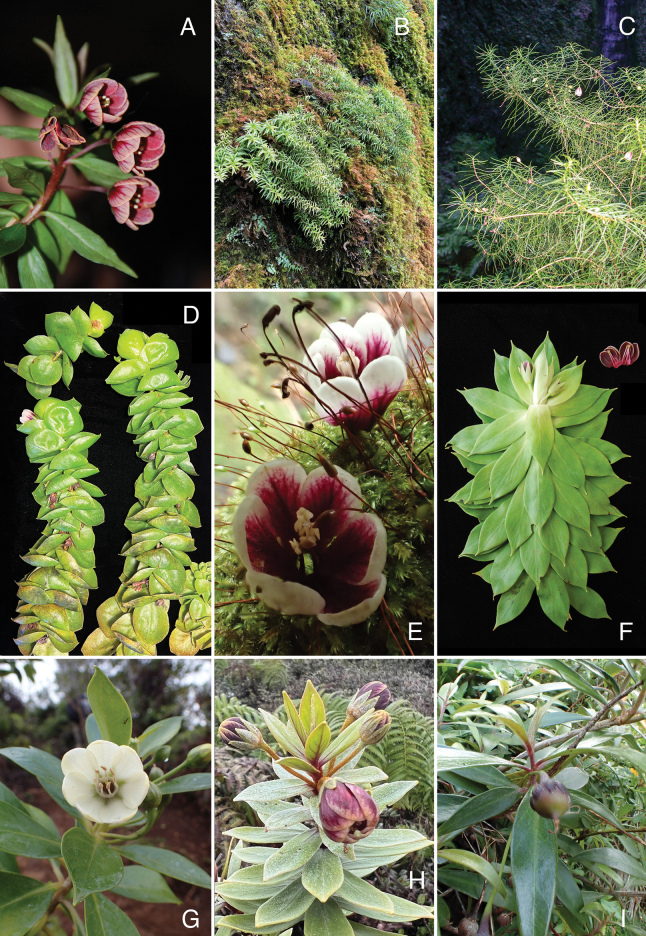
A. *Lysimachia hillebrandii*, erect with flower; B. *L. pendens*, pendent habit on wet cliff; C. *L. filifolia*, erect habit on stream bank; D. *L. iniki*, showing leaves cupped acroscopically; E. *L. iniki*, flowers fallen on moss with white corolla turning pink or purple towards base; F. *L. venosa*, with sessile leaves, purple corolla, and long calyx lobes; G. *L. glutinosa*, erect habit with cream corolla; H. *L. daphnoides* in bog habitat, erect habit and purple corolla; I. *L. scopulensis*, with purple calyx lobes and immature capsule. A. Field photo, 10 Jun 2010, Pi‘ina‘au, E Maui; B. Field photo, 14 Feb 2022, Blue Hole, Wailua, Kaua‘i; C. Field photo, 12 Jan 2008, Waikoko, Kaua‘i, *Wood 12774* (PTBG); D. Field photo, 9 Apr 2024, Blue Hole, Wailua, Kaua‘i, *Wood et al. 19481* (PTBG); E. Field photo, 21 Feb 2017, Blue Hole, Wailua, Kaua‘i, *Wood et al. 17263* (PTBG); F. Field photo, 11 Jan 2012, Kawaikini, Kaua‘i, *Wood 14845* (PTBG); G. Field photo, 30 Mar 2016, Kalalau, Kaua‘i; H. Field photo, 7 Nov 2024, Alaka‘i, Kaua‘i, *Wood et al. 19649* (PTBG); I. Field photo, 4 Feb 2016, Kalalau, Kaua‘i, *Wood et al. 16734* (PTBG).

Based on an analysis of the plastome phylogeny, *Lysimachia barcae* (represented by *Wood 16724* and *Wood 16725*) is most closely related to a Kaua‘i collection of *Lysimachia pendens* K.L.Marr (100% UFBoot support) and nested within a larger Kaua‘i clade, including *L. hillebrandii*, *L. filifolia* C.N.Forbes & Lydgate, *L. iniki* K.L.Marr and *L. venosa* (Wawra) H.St.John (Fig. 5A–F), with 99% UFBoot support. However, the topologies derived from nuclear (Lichter-Marck et al., in prep) and plastid DNA showed a high degree of conflict, potentially further complicated by polyploidy, as the only two published chromosome counts for Hawaiian *Lysimachia*, *L. glutinosa* Rock and *L hillebrandii* (Fig. 5A, G) are both hexaploid (*2n* = 72; Carr (1978); Kiehn (2005)). Other diverse Hawaiian endemic lineages have shown patterns of cytonuclear discordance (Dunbar‐Co et al. 2008; Yang et al. 2018; Knope et al. 2020; Baldwin et al. 2021), which may reflect chloroplast capture or ongoing hybridisation.

Morphologically, *Lysimachia barcae* is quite distinctive in comparison to all other currently described *Lysimachia* with its unique combination of mature stems villous to tomentose, petioles 1–3 mm long, leaves ovate, cordate to subcordate or rounded at base, with primary and secondary veins conspicuously prominent, purple-red, raised on abaxial surface, often densely hirsute to pilose and pedicels 35–70 mm long. It has some minor similarities to *L. hillebrandii* (Fig. 5A), with both being sprawling shrubs and having alternate leaves with some overlap in the length and width of leaves, yet *L. barcae* can be easily separated by features stated in the diagnosis. Superficially, the leaves of *L. barcae* have some overlap in length and width to the Moloka‘i species *L. maxima* (R.Knuth) H.St.John, yet differs from the latter species in having older stems villous to tomentose (vs. glabrate), leaves alternate, mostly ovate, with cordate to subcordate or rounded base (vs. leaves ternate, mostly obovate, with cuneate base) and pedicles 35–70 mm long (vs. 20–35 mm long). The unique cordate to subcordate or rounded leaf base of *L. barcae* has similarities to the cordate leaf base of *L. iniki* (Fig. 5D, E), yet differs from the latter species in having leaves ovate and flat or weakly revolute (vs. leaves obovate and cupped upwards), petioles 1–3 mm long (vs. sessile), stem tips non-viscid, tomentose (vs. viscid-hirsute), sprawling habit (vs. pendent), pedicels 35–70 mm long, pilose, pendulous (vs. 15–25 cm long, viscid-hirtellous, erect), calyx length 5–6 mm long (vs. 8–10 mm long) and corolla lobes purple with yellowish margin, 10–14 mm long (vs. white turning pink or purple towards base, 15–16 mm long) (see Table 1).

**Table 1.** Comparison of morphological characters for endemic Hawaiian *Lysimachia* on Kaua‘i (from Wagner et al. 1990, 1999, and Marr and Bohm 1997; and review of specimens at PTBG herbarium).

Species	L. barcae	L. daphnoides	L. filifolia	L. glutinosa	L. hillebrandii	L. iniki	L. kalalauensis	L. pendens	L. scopulensis	L. venosa
**Habit**	sprawling shrub	erect shrub	erect shrub	erect shrub	sprawling shrub	pendulous shrub	sprawling shrub	pendulous shrub	erect shrub	erect shrub
**Older stem pubescence**	villous	glabrate	glabrous	glabrous	glabrate	hirsute	glabrate	glabrate	glabrate	glabrous
**Stem tip pubescence**	tomentose	viscid-hirtellous	glabrous to sparsely pubescent	viscid-glabrous	tomentose	viscid-hirsute	tomentose	tomentose	pulverulent	glabrate
**Leaf shape**	ovate, rarely lanceolate	oblanceolate	linear-filiform	oblanceolate to elliptic	elliptic-ovate to elliptic-lanceolate	obovate, cupped upwards	elliptic	narrowly lanceolate	oblanceolate to obovate	oblanceolate
**Leaf length (mm)**	(35–)40–70	20–53	15–54	60–150	(22–)25–90	(35–)37–45(–54)	48–95	(20–) 25–30(–45)	(33–)55–65(–86)	60–90(–110)
**Leaf width (mm)**	(18–)20–45	6–14(–21)	0.3–1.8	15–45	(8–)10–45	(25–)35–38)(–42)	16–34	2–4	(5–)8–11(–23)	10–30
**Leaf base**	cordate to subcordate or rounded	attenuate	bluntly acute	attenuate	cuneate to attenuate	cordate	cuneate	attenuate	acute	truncate to attenuate
**Secondary leaf venation**	prominent	obscure	obscure	prominent to obscure	prominent to obscure	prominent	prominent to obscure	obscure	prominent	obscure
**Abaxial leaf pubescence**	hirsute to pilose	parts rusty viscid-hirtellous	glabrous	viscid-glabrous	pilose to glabrate	viscid-hirtellous to glabrate	pilose to glabrate	pilose	glabrous	glabrous
**Petiole length**	1–3 mm	sessile	sessile	sessile	4–10 mm	sessile	5–10 mm	1–2 mm	2–5 mm	sessile
**Pedicel length (mm)**	35–70	15–35(–110)	18–30(–60)	25–56(–75)	12–37(–45)	15–25	25–30(–45)	(6–)9–12(–14)	20–45	15–30
**Pedicel pubescence**	pilose	viscid-hirtellous	sparsely puberulent	viscid-glabrous	pilose	viscid-hirtellous	pilose	tomentose	glabrate	glabrate
**Pedicels erect or pendulous**	pendulous	erect	pendulous	erect	erect	erect	pendulous	erect	erect	erect
**Calyx length**	5–6 mm	7–9 mm	4–5.3(–6) mm	10–15 mm	4–8(–9) mm	8–10 mm	(9–)11–16 mm	4–6 mm	(2.5–)4–5 mm	13–19 mm
**Calyx colour**	green	green	green	green	green	green	green	green	green w/ purple-red base	green
**Calyx pubescence**	pilose to strigose	sparsely viscid-hirtellous	glabrous to glabrate	viscid-glabrous	pilose to tomentose	viscid-hirtellous	strigose	pilose to strigose	glabrate	glabrous, w/glabrate base
**Corolla lobe length**	10–14 mm	15–20 mm	6–10 mm	18–25 mm	10–20 mm	15–16 mm	16–18 mm	7–8.5 mm	7–11 mm	15–22 mm
**Corolla colour**	purple w/ margins yellowish	purple	purple	white	purple	white w/ pink or purple base	greenish w/purple-red base	red	red	purple w/ pale purple margins
**Capsule length**	6–9 mm	9–10 mm	5–6 mm	8–15 mm	8–9 mm	6–7 mm	8–10 mm	5–7 mm	6.5–7.5(–9) mm	9–11 mm

**Figure 6.**
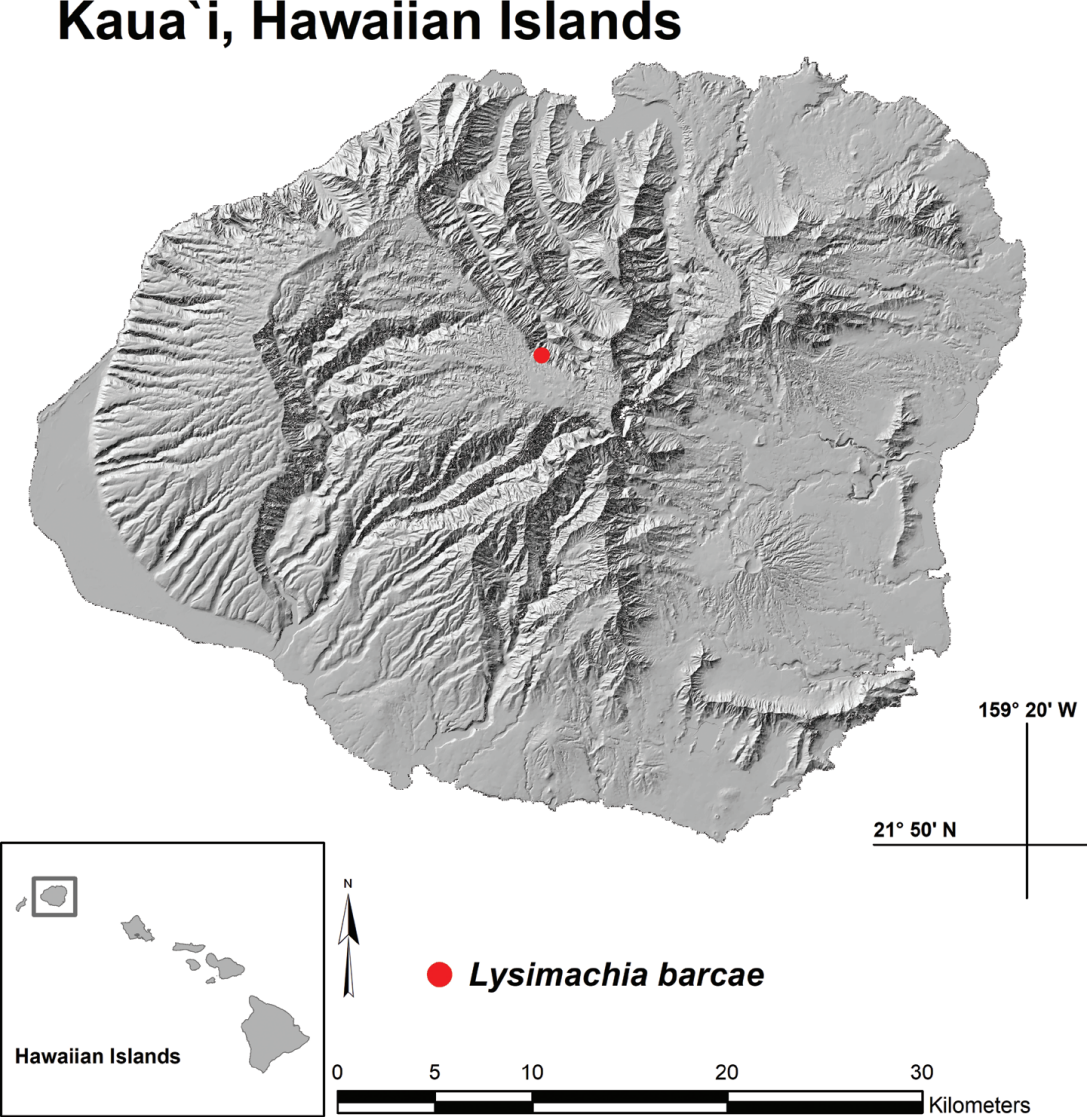
Distribution map with red dot indicating single known location of *Lysimachia barcae*, Wainiha Valley, Kaua‘i, Hawai‘i.

**Figure 7.**
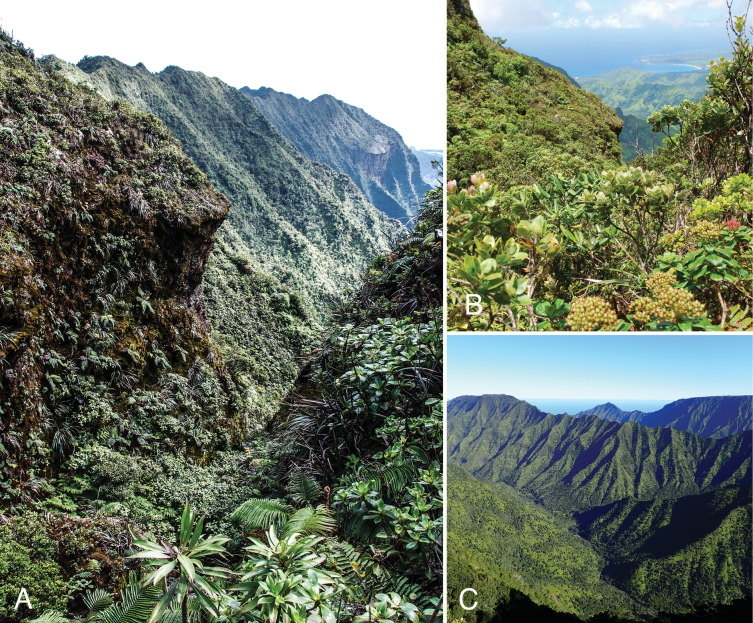
Montane wet forest habitat of *Lysimachia barcae*, showing steep slopes and ridges around the valley rim of Wainiha, Kaua‘i. Field photos: A. View NW from Wainiha rim with vertical cliffs of Pali‘ele‘ele in far distance, 30 July 2014; B. View NE from Wainiha rim with crescent bay of Hanalei in far distance, 30 July 2014; C. View ENE from Wainiha rim with Mahinakēhau Ridge in centre and Nāmolokama Plateau in far right distance, 19 Jan 2016.

**Distribution and ecology.***Lysimachia barcae* is narrowly endemic to the volcanic island of Kaua‘i where it has been documented around the north-central Alaka‘i plateau along a precipitously steep, moss-covered, talus-strewn ridge that descends into Wainiha Valley (Figs 6, 7). This new species is known from only a single colony of ten mature individuals at ca. 1150–1190 m elevation. Rainfall in the immediate vicinity of the type locality averages between 6000 and 7000 mm a year. The forest has a closed to open canopy and is dominated by canopy trees of *Metrosideros* Banks ex Gaertn. (Myrtaceae) and *Cheirodendron* Nutt. ex Seem. (Araliaceae). The associated genera of understorey trees, shrubs, herbs and vines are floristically rich and include endemic species of *Bidens* L., *Dubautia* Gaudich. (Asteraceae); *Clermontia* Gaudich. (Campanulaceae); *Perrottetia* Kunth (Dipentodontaceae); *Vaccinium* L. (Ericaceae); *Cyrtandra* J.R.Forst. & G.Forst. (Gesneriaceae); *Scaevola* L. (Goodeniaceae); *Hydrangea* Gronov. ex L. (Hydrangeaceae); *Geniostoma* J.R.Forst. & G.Forst. (Loganiaceae); *Myrsine* L. (Primulaceae); *Syzygium* Gaertn. (Myrtaceae); *Freycinetia* Gaudich. (Pandanaceae); *Peperomia* Giseke, (Piperaceae); *Coprosma* J.R.Forst. & G.Forst., *Kadua* Cham. & Schltdl., *Psychotria* L. (Rubiaceae); *Melicope* J.R.Forst. & G.Forst. (Rutaceae); and *Smilax* L. (Smilacaceae). Genera of sedges and grasses include *Carex* L., *Cyperus* L., *Machaerina* Vahl (Cyperaceae); and *Eragrostis* Wolf. (Poaceae). Genera of ferns include *Asplenium* L., *Hymenasplenium* Hayata (Aspleniaceae); *Deparia* Hook. & Grev., *Diplazium* Sw. (Athyriaceae); *Sadleria* Kaulf. (Blechnaceae); *Cibotium* Kaulf. (Cibotiaceae); *Microlepia* C.Presl (Dennstaedtiaceae); *Dryopteris* Adans. (Dryopteridaceae); *Dicranopteris* Bernh. (Gleicheniaceae); *Hymenophyllum* Sm. (Hymenophyllaceae); *Adenophorus* Gaudich. (Polypodiaceae); and *Hoiokula* S.E.Fawc. & A.R.Sm. (Thelypteridaceae). Wood (2007) reports high levels of floristic diversity in the greater Wainiha/Alaka‘i Region of Kaua‘i with ca. 266 native vascular plant taxa, including 92 single-island endemics.

##### ﻿Key to Hawaiian *Lysimachia*

The following key to Hawaiian *Lysimachia* has been modified from Wagner et al. (1990, pp. 1079–1080) to accommodate *Lysimachia barcae*, along with *Lysimachia iniki*, *L. pendens* and *L. scopulensis* K.L.Marr, which were discovered and described post Wagner et al. (1990).

Note: HI = Hawaiian Islands; H = Hawai‘i (Big Island); K = Kaua‘i; L = Lāna‘i; M = Maui; Mo = Moloka‘i; Ni = Ni‘ihau; O = O‘ahu.

**Table:** 

1	Capsules irregularly dehiscent; somewhat fleshy perennial herbs; leaves spatulate to elliptic; flowers in racemes; Ni, K, Mo, M, H (and scattered localities outside HI)	***L. mauritiana***
–	Capsules regularly dehiscent, 5–10-valved; shrubs; leaves linear, obovate, oblanceolate, elliptic or broadly ovate; flowers solitary or rarely up to 3 in the leaf axils	**2**
2	Leaves narrowly lanceolate or linear-filiform 0.3–5 mm wide	**3**
–	Leaves linear-elliptic to broadly elliptic, oblanceolate or ovate, 5–100 mm wide	**5**
3	Leaves narrowly lanceolate, pubescent; pedicels 9–12 mm long; K	***L. pendens***
–	Leaves linear-filiform, glabrous to glabrate; pedicels 15–35(–60)	**4**
4	Young stems rusty pilose to tomentose; leaves 1–5 mm wide, petioles 3–8(–15); pedicels erect; corolla 9–11 mm long; Mo, M	***L. remyi*** Hillebr.
–	Young stems glabrous to sparsely pubescent; leaves 0.3–1.8 mm wide, sessile; pedicels pendulous; corolla 6–10 mm long; K, O	***L. filifolia***
5	Calyx 20–28(–31) mm long; corolla lobes erose; plants pubescent with purple hairs; leaf venation sharply contrasting purple to purplish-brown on lower surface; O	***L. forbesii***
–	Calyx 4–19 mm long; corolla lobes entire; plants pubescent with rusty brown hairs or glabrous; leaf venation not sharply contrasting in colour on lower surface	**6**
6	Corolla cream or white turning pink or purple towards base, 15–25 mm long	**7**
–	Corolla dark purple to reddish-purple, burgundy, or green with purplish-red base, 9–20 mm long	**8**
7	Stems glabrous; corolla cream, 18–25 mm long; K	***L. glutinosa***
–	Stems densely hirsute; corolla white turning pink or purple towards base, 15–16 mm long; K	***L. iniki***
8	Mature leaves densely rusty tomentose on lower surface, upper surface appressed rusty pilose; M	***L. lydgatei* Hillebr.**
–	Mature leaves glabrous, glabrate, hirsute, or pilose, but not tomentose	**9**
9	Leaves broadest above middle, oblong-oblanceolate, oblanceolate to obovate or elliptic-obovate, sessile or in *L. scopulensis* with petioles 3–4.5 mm long and leaves pulverulent	**10**
–	Leaves broadest at or below middle, oblong-linear, linear-elliptic, narrowly to broadly elliptic, elliptic-lanceolate to broadly elliptic-ovate, petioles (1–)3–10 mm long; leaves not pulverulent	**13**
10	Leaves ternate; Mo	***L. maxima***
–	Leaves alternate	**11**
11	Young stems and leaves pulverulent, calyx lobes green with purple-red base, widely ovate; K	***L. scopulensis***
–	Young stems and leaves not pulverulent, calyx lobes green, narrowly lanceolate to narrowly ovate	**12**
12	Leaves 20–53 mm long, 6–14(–21) mm wide; calyx lobes 7–9 mm long; K	***L. daphnoides* (A.Gray) Hillebr.**
–	Leaves 60–110 mm long, 10–30 mm wide; calyx lobes 12–19 mm long; K	***L. venosa***
13	Leaves oblong-linear or linear-elliptic to narrowly elliptic, often somewhat falcate, 5–8 mm wide, occasionally some of the main stem leaves ovate, up to 15 mm wide; corolla 9–11 mm long; Mo, M	***L. remyi***
–	Leaves ovate, broadly elliptic-ovate to elliptic-lanceolate or narrowly to broadly elliptic, (8–)10–45 mm wide; corolla 10–20 mm long	**14**
14	Older stems villous to tomentose; petioles 1–3 mm long, leaf base cordate to subcordate or rounded; pedicels 35–70 mm long; K	***L. barcae* sp. nov.**
–	Older stems glabrate; petioles 4–10 mm long, leaf base cuneate to attenuate; pedicels 12–45 mm long	**15**
15	Calyx lobes broadly ovate to lanceolate, 4–8(–9) mm long; corolla reddish-purple to burgundy; K, O, Mo, L, M	***L. hillebrandii***
–	Calyx lobes narrowly lanceolate, (9–)11–16 mm long; corolla greenish with purple-red base; K	***L. kalalauensis* Skottsb.**

##### ﻿Key to endemic Hawaiian *Lysimachia* on Kaua‘i

**Table:** 

1	Leaves narrowly lanceolate or linear-filiform 0.3–4 mm wide	**2**
–	Leaves linear-elliptic to broadly elliptic, oblanceolate or ovate, 5–45 mm wide	**3**
2	Leaves narrowly lanceolate, 2–4 mm wide; calyx lobes narrowly ovate	***L. pendens***
–	Leaves linear-filiform 0.3–1.8 mm wide; calyx lobes narrowly lanceolate	***L. filifolia***
3	Corolla cream or white turning pink or purple towards base; young parts viscid	**4**
–	Corolla purple, dark red or green with purple base; young parts not viscid, except in *L. daphnoides*	**5**
4	Pendulous shrubs; stems densely hirsute; leaves cupped acroscopically, obovate to orbicular, base cordate; corolla white turning pink or purple towards base, 15–16 mm long	***L. iniki***
–	Erect shrubs; stems glabrous; leaves not cupped, oblanceolate to elliptic, base attenuate; corolla cream, 18–25 mm long	***L. glutinosa***
5	Young stems pulverulent; calyx lobes purple, sometimes green with purple-red base, widely ovate; K	***L. scopulensis***
–	Young stems not pulverulent; calyx lobes green, narrow to broadly lanceolate or narrowly ovate	**6**
6	Older stems villous; leaf base cordate to subcordate or rounded	***L. barcae***
–	Older stems glabrous to glabrate; leaf base cuneate, attenuate or truncate	**7**
7	Erect shrubs; leaves sessile, oblanceolate	**8**
–	Sprawling shrubs; petioles 4–10 mm, leaves not oblanceolate	**9**
8	Young stems viscid, hirtellous; leaves 20–53 mm long, hirtellous; pedicels hirtellous; calyx 7–9 mm long, hirtellous	***L. daphnoides***
–	Young stems not viscid, glabrate; leaves 60–110 mm long, glabrous; pedicels glabrate; calyx 13–19 mm long, glabrous to glabrate	***L. venosa***
9	Calyx lobes broadly ovate to lanceolate, 4–8(–9) mm long; capsules 8–9 mm long; corolla reddish-purple to burgundy	***L. hillebrandii***
–	Calyx lobes narrowly lanceolate, (9–)11–16 mm long; capsules 9–10 mm long; corolla greenish with purple-red base	***L. kalalauensis***

##### ﻿Preliminary conservation assessment. IUCN Red List Category

*Lysimachia barcae* falls into the Critically Endangered (CR) category according to the IUCN criteria (B1ab(iii)+2ab(iii)+D) which reflects an EOO 1 km^2^ and AOO of 1 km^2^, a continuing decline in the quality of habitat and a population size estimated to number fewer than 50 mature individuals. It is well known that invasive plant and animal species threaten nearly all native flora throughout the Hawaiian Islands and *Lysimachia barcae* is no exception. The continued decline in quality of surrounding habitat where *L. barcae* occurs is evidenced by degradation from invasive non-native mammals such as pigs (*Sus scrofa* L.), rats (*Rattus* spp.) and black-tailed deer (*Odocoileus hemionus columbianus* Richardson), in addition to the dense infestations of non-native plant species such as *Erigeron karvinskianus* DC. (Asteraceae) and *Hedychium gardnerianum* Sheph. ex Ker Gawl. (Zingiberaceae) which outcompete and suppress native bryophytes, seedlings and sporophytes through aggressive colonisation and shading. Additionally, *Hedychium gardnerianum* has established extensive rhizomatous networks that form dense, monotypic stands, to the extent that native habitats are unable to establish or persist. *Lysimachia barcae* is also highly susceptible to stochastic events such as landslides, which could result in significant habitat loss or population extirpation.

The lack of biotic regional data has become a serious barrier to sound management of the Earth’s remaining natural ecosystems and we hope that this formal description will stimulate future conservation and exploratory efforts to locate more colonies of *Lysimachia barcae*. There is still a large expanse of suitable, unexplored habitat around Wainiha’s rugged and highly variable physical geography (Fig. 7) and we predict that additional colonies of *L. barcae* will likely be found.

Seeds and cuttings of *Lysimachia barcae* have been collected by the Plant Extinction Prevention Program (PEPP) and the National Tropical Botanical Garden (NTBG) Science staff and plants are currently being cultivated by the Hawaii State Division of Forestry and Wildlife (DOFAW) nursery, Kaua‘i, Hawai‘i.

We now estimate that over 130 Hawaiian vascular plant taxa have become extinct to date (Wood et al. 2019) and the urgency to stem the extinction crisis becomes clearer when we realise that a significant number of endemic Kaua‘i taxa are limited to very small numbers, within a single valley or mountain range, with barely enough individuals to maintain viable populations for their long term survivorship. This, along with the understanding that each species is a masterpiece of evolution, extending back millions of years, profoundly demonstrates the urgency to support active conservation efforts and advance horticultural science for their cultivation.

## Supplementary Material

XML Treatment for Lysimachia barcae

## ﻿Additional information

### Use of AI

No use of AI was reported.

### Author ORCIDs

Kenneth R. Wood https://orcid.org/0000-0001-6446-1154

Warren L. Wagner https://orcid.org/0000-0001-5012-8422

Susan Fawcett https://orcid.org/0000-0001-5021-1779

Scott Heintzman https://orcid.org/0009-0002-8580-7630

### Data availability

Scripts used for data analyses are available on GitHub github.com/susanfawcett/Hawaii.
